# Focusing on the Catalysts of the Pd- and Ni-Catalyzed Hirao Reactions

**DOI:** 10.3390/molecules25173897

**Published:** 2020-08-26

**Authors:** György Keglevich, Réka Henyecz, Zoltán Mucsi

**Affiliations:** Department of Organic Chemistry and Technology, Budapest University of Technology and Economics, 1521 Budapest, Hungary; henyecz.reka@mail.bme.hu (R.H.); zoltanmucsi@gmail.com (Z.M.)

**Keywords:** Hirao reaction, P–C coupling, Pd-catalyst, Ni-catalyst, catalyst formation, mechanism, ligation

## Abstract

The Hirao reaction involving the phosphinoylation or phosphonation of aryl halides by >P(O)H reagents is a P–C bond forming transformation belonging to the recently very hot topic of cross-couplings. The Pd- or Ni-catalyzed variations take place via the usual cycle including oxidative addition, ligand exchange, and reductive elimination. However, according to the literature, the nature of the transition metal catalysts is not unambiguous. In this feature article, the catalysts described for the Pd(OAc)_2_-promoted cases are summarized, and it is concluded that the “(HOY_2_P)_2_Pd(0)” species (Y = aryl, alkoxy) is the real catalyst. In our model, the excess of the >P(O)H reagent served as the P-ligand. During the less studied Ni(II)-catalyzed instances the “(HOY_2_P)(−OY_2_P)Ni(II)Cl^−^” form was found to enter the catalytic cycle. The newest conclusions involving the exact structure of the catalysts, and the mechanism for their formation explored by us were supported by our earlier experimental data and theoretical calculations.

## 1. Introduction

### 1.1. Palladium-Catalyzed Hirao Reactions

An up-to-date means for the preparation of dialkyl arylphosphonates is the Hirao P–C coupling between a dialkyl phosphite and an aryl bromide using Pd(PPh_3_)_4_ as the catalyst, and advantageously, triethylamine as the base in a wide range of solvents [1,2,3,4,5,6,7,8,9]. To date, a number of variations of the P–C couplings were developed comprising in situ formed catalysts from suitable precursors, such as Pd(OAc)_2_ and PdCl_2_, and different added mono- or bidentate P-ligands. The protocol was extended to *H*-phosphinates, as well as secondary phosphine oxides applying a wide range of arenes [10,11,12,13,14,15,16,17,18,19,20,21,22]. Later on, Ni- and Cu complexes were also used as catalysts [10,11,23,24,25,26,27,28,29,30,31]. Green chemical approaches, including microwave (MW)-assistance [32,33,34,35,36,37] and phase transfer catalysis, were also elaborated [38,39,40,41].

Regarding Pd catalysis, either Pd(PPh_3_)_4_, or Pd(OAc)_2_ together with a mono and bidentate P-ligand may be utilized in the P–C couplings under discussion. Keglevich with co-workers elaborated a MW-assisted version of the Hirao reaction, when Pd(OAc)_2_ or NiCl_2_ was used without the P-ligands applied earlier [42,43,44,45,46,47]. This variation may be called a “P-ligand-free” Hirao reaction [42,43,44,45,46,47]. However, the reality is that, in these cases, the excess of the >P(O)H reagent serves as the reducing agent, and as the P-ligand. The “P-ligand-free” method has the advantage that there is no need for expensive and air-sensitive P-ligands, hence the costs and environmental burdens may be decreased. Moreover, the realization of the P–C coupling reaction is simplified as a single >P(O)H species may serve as the reagent, the reducing agent and the P-ligand at the same time.

**Figure 1:**
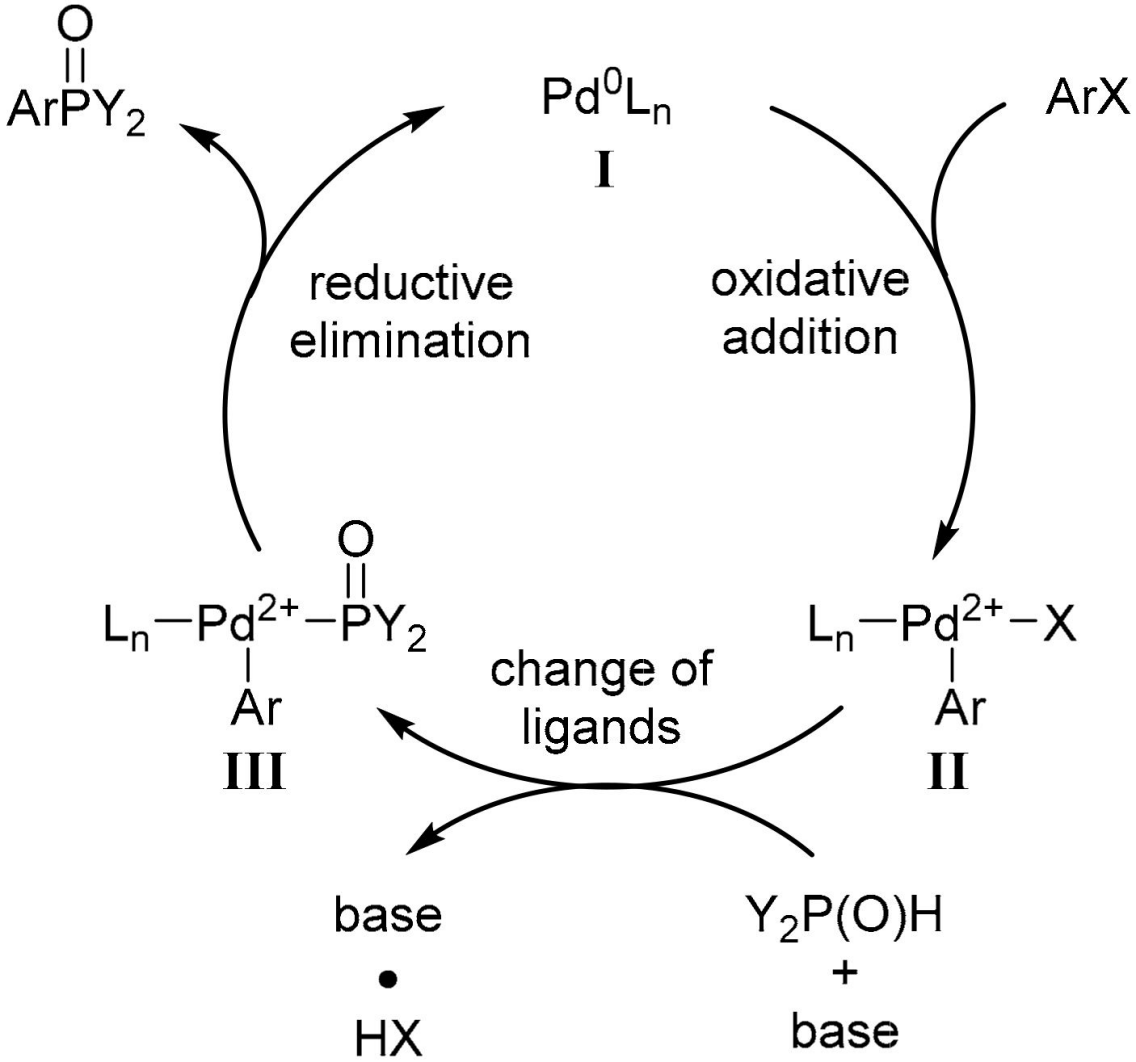
General scheme for the Pd-catalyzed Hirao reaction.

The catalytic cycle for the Pd(0)-catalyzed P–C coupling reaction is presented in Figure 1. The main steps are the oxidative addition of the aryl halide to the Pd(0) complex (**I**) to afford Pd(II) complex **II**, the change of ligands leading to key intermediate **III**, and the reductive elimination resulting in the formation of the final product (ArP(O)Y_2_, Y = aryl [Ar] or alkoxy), and regenerating the Pd(0) catalyst [13,15,48,49,50,51,52].

**Scheme 1:**
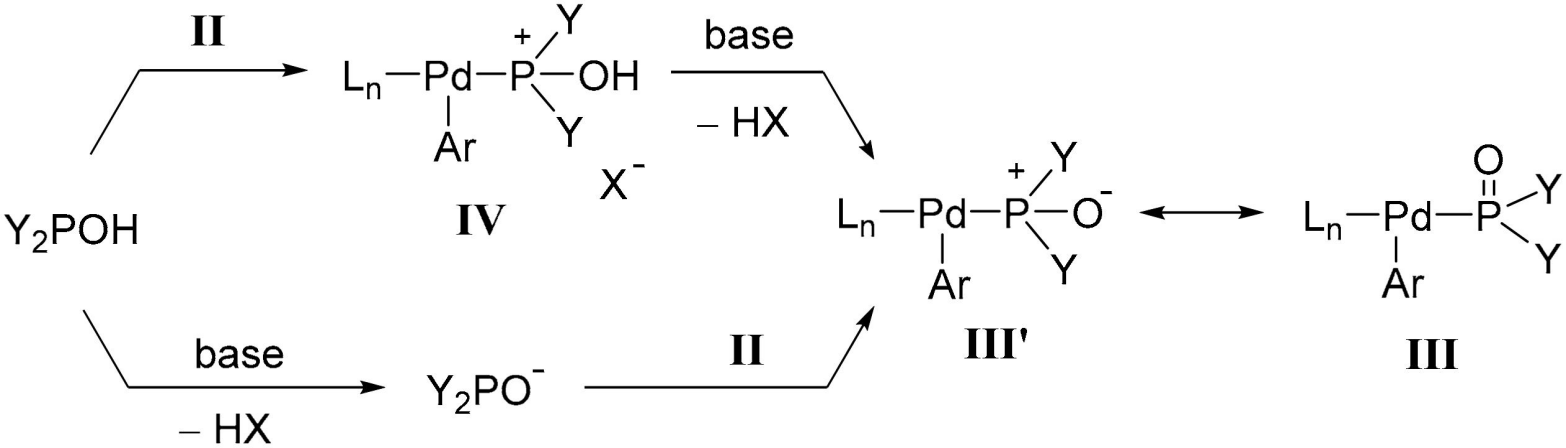
Refinement of the **II**→**III** conversion during the cycle of the Hirao reaction.

The oxidative addition is the rate determining step in almost all cross-coupling reactions [51]. The elemental steps are influenced by the nature of the aryl substrates, catalyst, and the solvent applied. The rate of the oxidative addition of aryl halides follows the reactivity order I > Br > Cl, and electron withdrawing substituents in the aromatic ring may facilitate the formation of the metal–carbon bond. The more electron-rich the ligands are, and, in general, the more polar the solvent is, the faster complex **II** is formed [50]. A few refinements of the ligand exchange were proposed by several authors. Such is the incorporation of the P(III) tautomeric form of the >P(O)H species in the primary Pd-adduct (**II**) to form intermediate **IV** undergoing a deprotonation by the base present in the mixture to furnish the secondary Pd-adduct (**III’**/**III**) (Scheme 1) [15,49,52]. Species **IV** may be deprotonated by weak tertiary amines. If a more acidic >P(O)H reagent and a stronger inorganic base (pK_a_ ≥ 18) are used, the Y_2_POH species may be first deprotonated, and then the anion Y_2_PO^–^ so formed enters the cycle resulting in the formation of complex **III’**. However, presence of the anion has not yet been detected [15,50,52].

Montchamp and co-workers applied solvent additives (e.g., ethylene glycol or dimethoxyethane) to facilitate the P–C coupling of *H*-phosphinates [15,52,53]. According to them, on the one hand, the co-solvent promotes the ligand exchange by taking part in the conversion of the pentavalent Y_2_P(O)H to the Y_2_POH tautomer, on the other hand, it stabilizes the Pd-catalyst. The rate of the last step is also influenced by the steric and electronic properties of the ligands involved in complex **III**. A c*is* geometry of the aryl group and the *P*-moiety is required for the reductive elimination to occur. Unlike other C–heteroatom cross-coupling reactions, the elimination of the P–C coupled product may be promoted by electron-donating substituents [54]. In case of bidentate ligands, the so-called “bite angle” may also affect the reaction rate: a larger angle induce faster elimination [55].

Kalek and Stawinski found that the addition of ionic additives, mainly acetates, had a positive effect on the course of the P–C couplings regarding reaction time [13,16,50]. According to the studies, acetate ions play role in all stages of the catalytic cycle: a more active catalyst complex may be formed (see later) [13], and the presence of ions accelerates the ligand exchange and the reductive elimination as well [50].

### 1.2. Nickel-Catalyzed P–C Coupling Reactions

Nickel catalysis is also often applied in P–C cross coupling reactions. On the one hand, reductive procedures involving Ni(II) salts together with Zn or Mg as the reductant were described [23,24,56], or Ni(0)(cod)_2_ was applied as the catalyst precursor [25,26,57,58,59], on the other hand, the Ni salts were used without reductive agents [60,61,62,63,64]. It is worth noting that in the above reactions, especially in the reductive variations, 2,2’-bipyridine was applied as the ligand. It was assumed that Ni(II) is reduced to Ni(0), and the bipyridine complex of the reduced form takes part in the oxidative addition of the aryl or other halide. This is followed by ligand exchange including the entry of the >P(O)H reagent. The last step is the reductive elimination [65]. Hence, regarding the redox background, a similar process was substantiated as for the Pd-case. However, no convincing evidence was presented for the Ni(0) → Ni(II) supposition at the start of the cycle.

Moreover, the way how the P–C couplings applying Ni(II) salts take place in the absence of reductants has not been investigated for long [60,61,62,63,64].

As it was already mentioned, the Keglevich group developed a MW-assisted version of the P–C coupling, and it was possible to apply NiCl_2_ instead of Pd(OAc)_2_. In these cases, there was no need to apply the usual P-ligands, instead, the excess of the >P(O)H reagent served as the ligand [47].

## 2. Catalysts for the Pd-Promoted P–C Couplings

### 2.1. General Considerations

**Figure 2:**
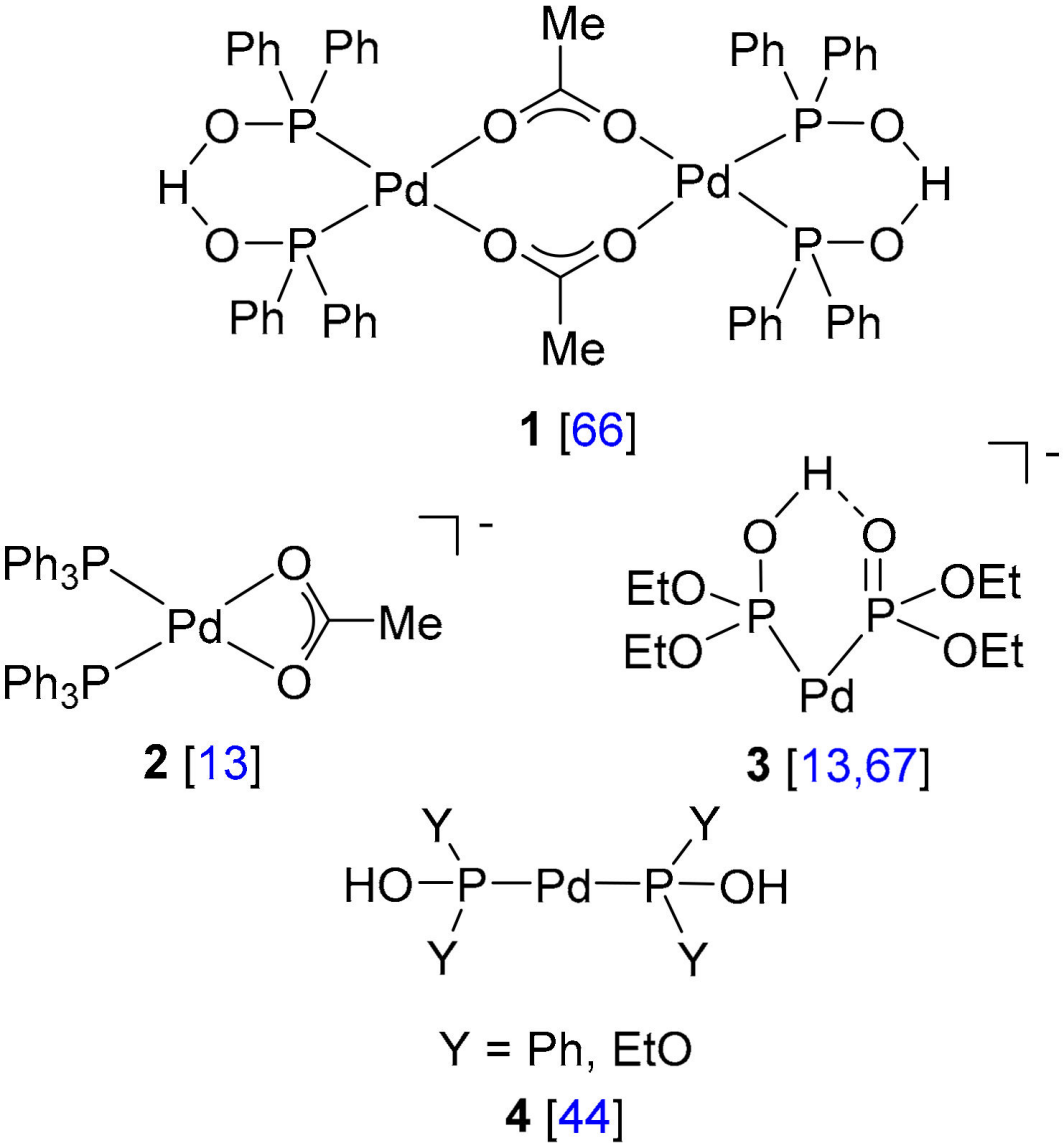
Pd complexes assumed in the Hirao reaction [13,44,66].

Let us survey the possible Pd complexes that were considered as catalysts in the Hirao reaction (Figure 2). All agreed on that regarding the palladium salt precursors, Pd(OAc)_2_ is the best choice due to its lipophilicity [10,12]. Although this salt consists of trimers or even polymers, its structure is generally represented as Pd(OAc)_2_. Buono et al. assumed the formation of the [{H(OPh_2_P)_2_}PdOAc]_2_ complex (**1**) in the interaction of Pd(OAc)_2_ and diphenylphosphine oxide [66]. The investigations of Amatore and Jutand on C–C coupling reactions showed that the interaction of Pd(OAc)_2_ and triphenylphosphine leads to (Ph_3_P)_2_Pd(OAc)_2_ complex, that is then converted to species **2** by reduction and the departure of an AcO^–^ ion [67]. Kalek and Stawinski also proposed (Ph_3_P)_2_PdAcO^–^ complex **2** as an active species in the P–C couplings involving Pd(OAc)_2_ as the catalyst precursor and PPh_3_ as the ligand [13]. At the same time, complex **3** was considered by them as an inactive species in the catalytic cycle [13], regardless that Ackermann et al. supposed a similar species (the deprotonated version of **4**) as an active catalyst in another kind of reaction, in the intermolecular α-arylation of 2-acylamino-chloroarenes to provide different oxindoles [68]. We suggested the simple “PdP_2_^”^ type complex (**4**) as the real catalyst in the Hirao reaction on the basis of our experiments and theoretical calculations [44].

### 2.2. Formation of the PdP_2_ Catalyst—Theoretical and Experimental Results

**Scheme 2:**
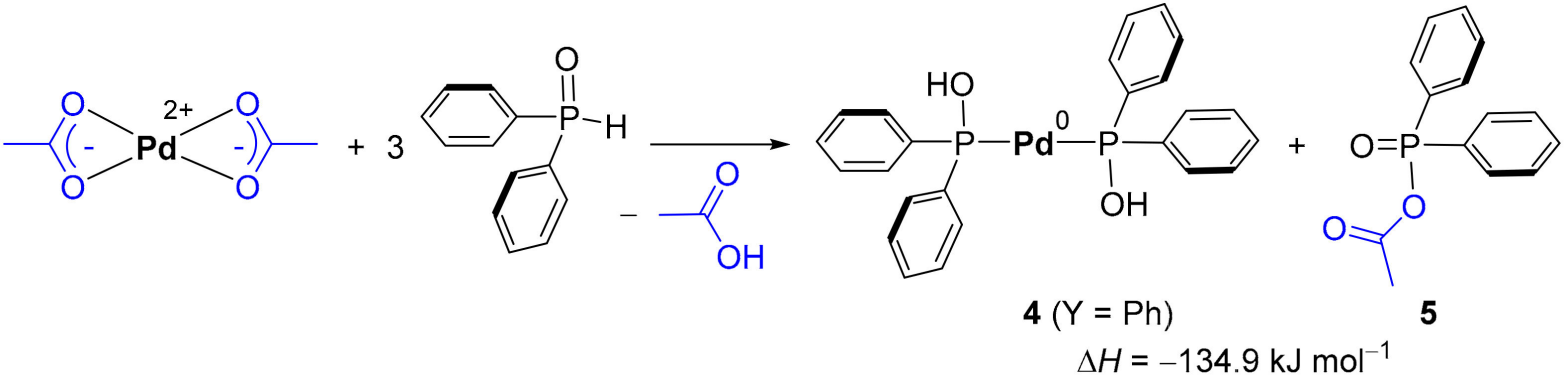
Thermodynamics for the reaction of Pd(OAc)_2_ with Ph_2_P(O)H affording [(HO)Ph_2_P]_2_Pd complex. The reaction enthalpy was calculated at the B3LYP level of theory applying 6–31G(d,p) for CHPO, and SDD(MWB28) for Pd.

We were the first who studied the formation of Pd complex **4** from Pd(OAc)_2_ and the excess of the Y_2_P(O)H reagent. Our observation was that in the “P-ligand-free” Hirao reaction, 1.3 equivalents of the >P(O)H reagent should be used if the Pd(OAc)_2_ catalyst is applied in a quantity of 10 mol% (three equivalents are needed for the catalyst, 1 equivalent as the reducing agent, two equivalents as the P-ligand) [42,43]. Complex [HO(EtO)_2_P]_2_Pd (**4**, Y = EtO) was prepared by us in a separate experiment reacting Pd(OAc)_2_ with 1.3 equivalents of (EtO)_2_P(O)H at 120 °C for 30 min. ^31^P NMR shift of the Pd complex (**4**, Y = EtO) was in agreement with that reported by Kalek et al. [13]. This complex was then tested in the reaction of (EtO)_2_P(O)H with PhBr carried out as elaborated by us [44]. Diethyl phenylphosphonate was obtained in a yield of 72% proving that the in situ formed catalyst is identical to species **4** (Y = EtO). It was also proved by us [44] that the interaction of Pd(OAc)_2_ and diphenylphosphine oxide furnishes a complex [(HO)Ph_2_P]_2_Pd (**4**, Y = Ph). Pd(II) was reduced to Pd(0), while the oxidation is manifested in by-product **5**. The formation of this species along with the energetics are shown in Scheme 2 [69].

**Scheme 3:**
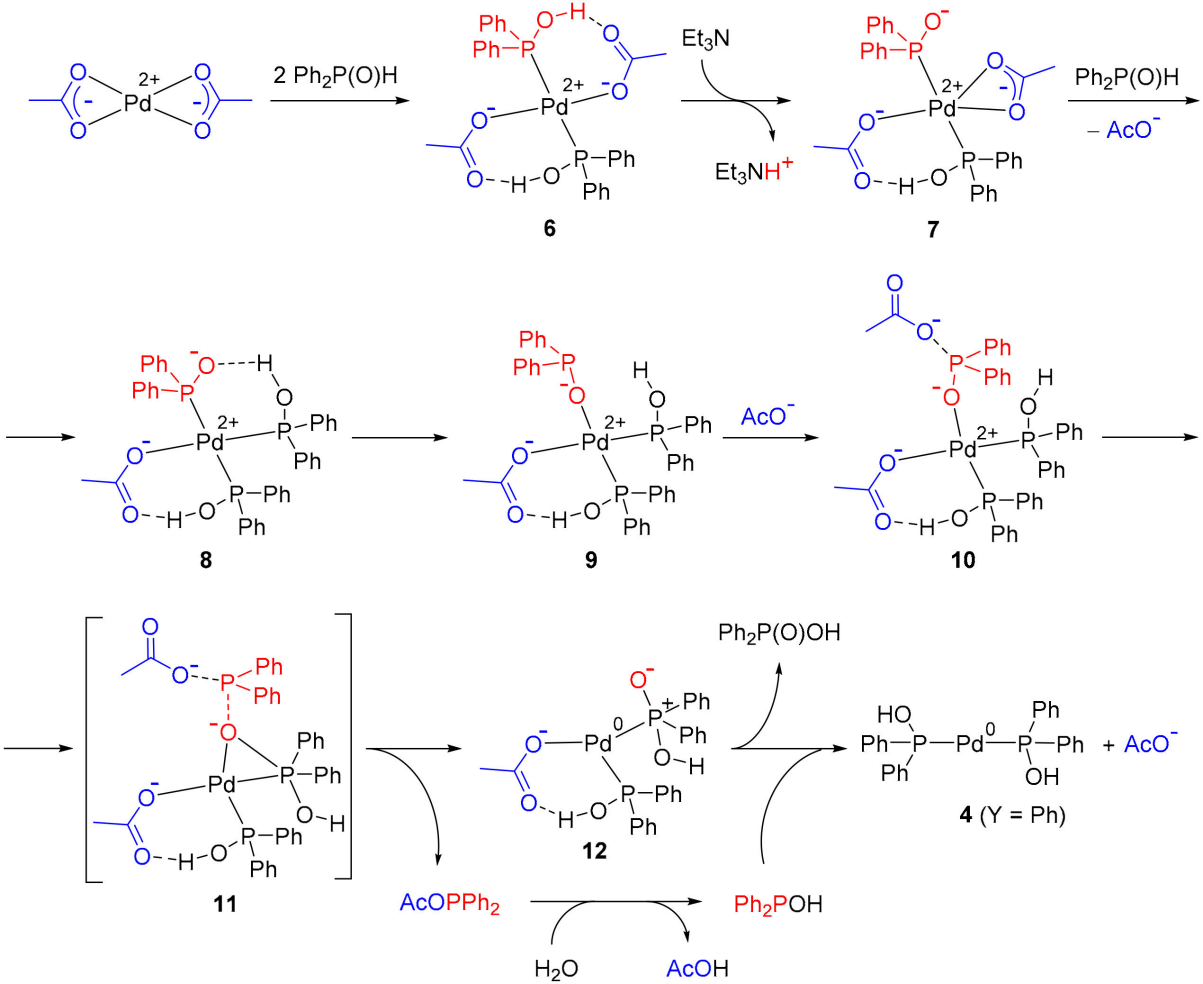
Fine mechanism for the formation of catalyst [(HO)Ph_2_P]_2_Pd from Pd(OAc)_2_ and Ph_2_P(O)H calculated by the B3LYP/genecp//PCM method using the 6–31G(d,p) basis set for CHOP, and SDD(MWB28) for Pd atoms including the explicit–implicit solvent model.

The mechanism of the catalyst formation was evaluated by theoretical calculations [69]. According to the general consideration and our earlier findings [44], the active oxidation state of Pd was assumed to be zero. As it was mentioned above, diphenylphosphine oxide serves as the reductive agent. The most probable reaction mechanism is presented in Scheme 3. The reaction of Pd(OAc)_2_ with two molecules of Ph_2_P(O)H affords to Pd(II) complex **6**, the deprotonation of which leads to species **7**. Then, the acetate anion of the “PdOAc” moiety is replaced by a third Ph_2_P(O)H molecule affording complex **8**. Then, an intermolecular *O*-insertion into the P–Pd bond of intermediate **8** gives species **9**. In the next step, an acetate anion is connected to the P atom of the “Ph_2_POPd” moiety of complex **9** to furnish species **10**. A reductive elimination from intermediate **10** via TS **11** provides complex **12**. A final stabilization of species **12** by the elimination of Ph_2_P(O)OH and AcO^−^, and by the simultaneous incorporation of the Ph_2_POH species formed by regeneration via hydrolysis of acyloxyphosphine by-product results in the formation of complex **4** (Y = Ph) that is the active catalyst [44].

**Scheme 4:**
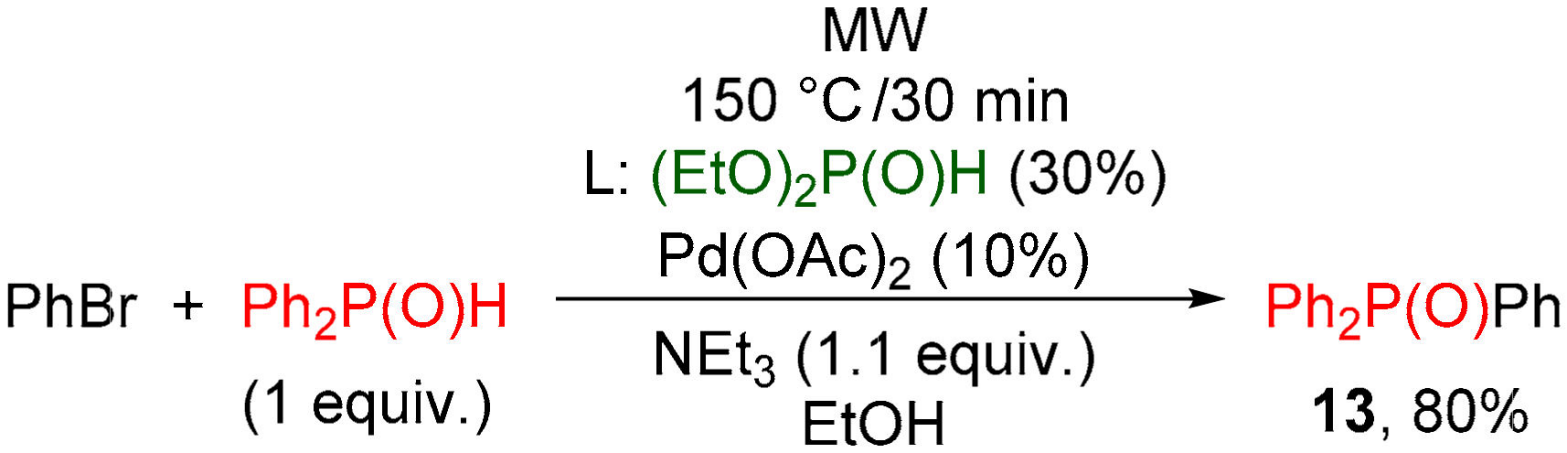
The Pd-catalyzed Hirao reaction of bromobenzene and diphenylphosphine oxide in the presence of diethyl phosphite as the reducing agent and the ligand.

We found that it is possible to use (EtO)_2_P(O)H as the reducing agent and as the P-ligand for the Pd formed in the P–C coupling of PhBr and Ph_2_P(O)H. The Hirao reaction carried out in the presence of 1 equivalent of Ph_2_P(O)H and 0.3 equivalents of (EtO)_2_P(O)H afforded Ph_3_PO (**13**) in a yield of 80% (Scheme 4) [44].

**Scheme 5:**
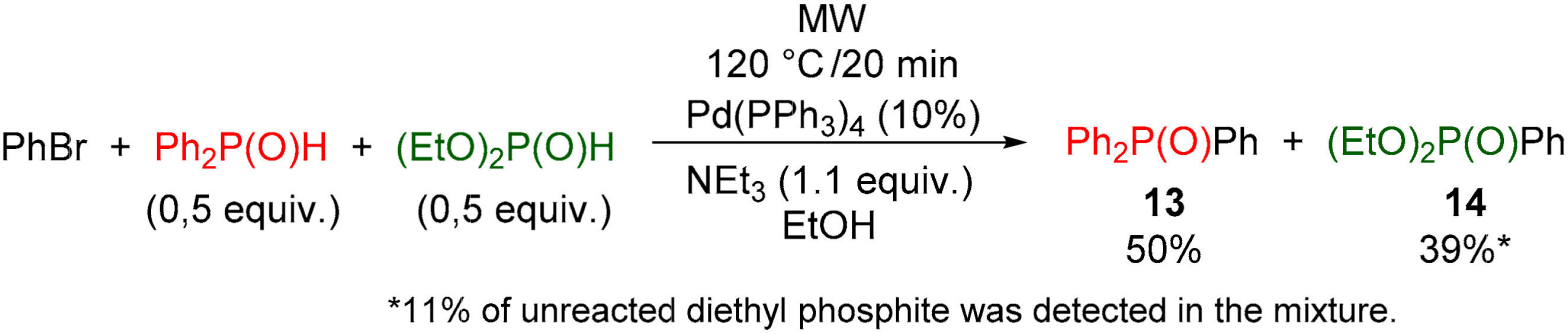
The concurrent P–C coupling of bromobenzene with the equimolar mixture of diphenylphosphine oxide and diethyl phosphite in the presence of Pd(PPh_3_)_4_.

The comparison of the reactivity of (EtO)_2_P(O)H and Ph_2_P(O)H is not easy, as there are three independent and consecutive reaction steps: reduction of the Pd(II) to Pd(0) (1), ligation of the Pd(0) so formed (2), and the intrinsic P–C coupling itself (3). As regards to the gross reactivity, (EtO)_2_P(O)H seems to be more reactive than Ph_2_P(O)H, if the reaction times required at 120 °C are compared. To be able to decide on the reactivity of the >P(O)H species in the P–C couplings, concurrent reactions comprising 0.5 equivalents of both Ph_2_P(O)H and (EtO)_2_P(O)H to one equivalent of bromobenzene were performed in the presence of 10 mol% of Pd(PPh_3_)_4_ at 120 °C, and interrupted before completion that suggested that Ph_2_P(O)H is slightly more reactive, than (EtO)_2_P(O)H in the P–C coupling reaction itself (Scheme 5) [44].

**Scheme 6:**
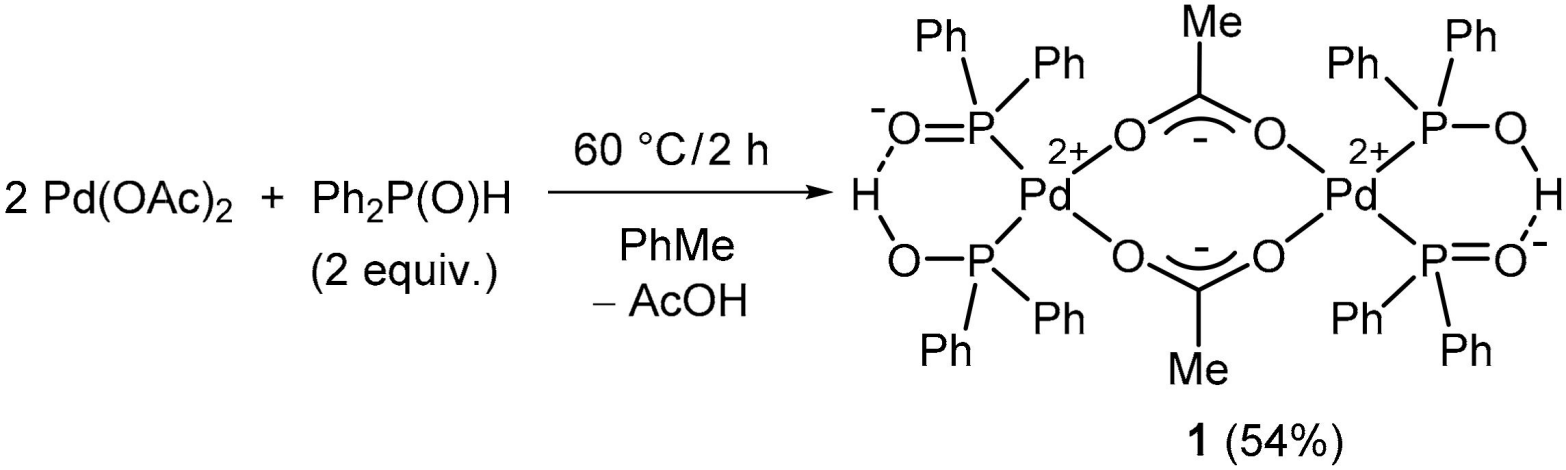
Assumed formation of bis(Pd complex) **1** from Pd(OAc)_2_ and Ph_2_P(O)H.

Returning to the idea of Buono et al. they claimed the formation of another kind of complex from the interaction of Pd(OAc)_2_ with a series of secondary phosphine oxides applied in a two equivalents quantity. The outcome is shown on the example of Ph_2_P(O)H as the P-reagent (Scheme 6) [66]. However, the structure of complex **1** was not confirmed.

We believe that in the latter case not complex **1**, but the [(HO)Ph_2_P]_2_Pd complex (**4**, Y = Ph) was formed.

**Scheme 7:**
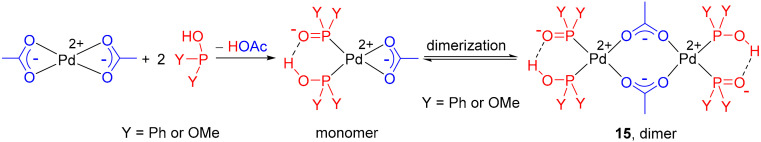
Theoretical study on the formation of monomeric and dimeric Pd(II) complexes from Pd(OAc)_2_ and Y_2_POH calculated at the B3LYP/6-31G(d,p)//PCM(MeCN) level.

**Scheme 8:**
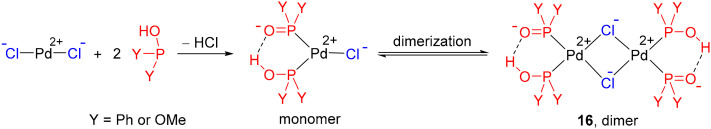
Theoretical study on the formation of monomeric and dimeric Pd(II) complexes from PdCl_2_ and Y_2_POH calculated at the B3LYP/6-31G(d,p)//PCM(MeCN) level.

Comparative theoretical calculations suggested that while complex **15** is not a stable species (Scheme 7), the dimer-like complex with two chloride anions (**16**) may exist (Scheme 8).

### 2.3. A Theoretical Study on the Ligation of Pd(0)

**Scheme 9:**
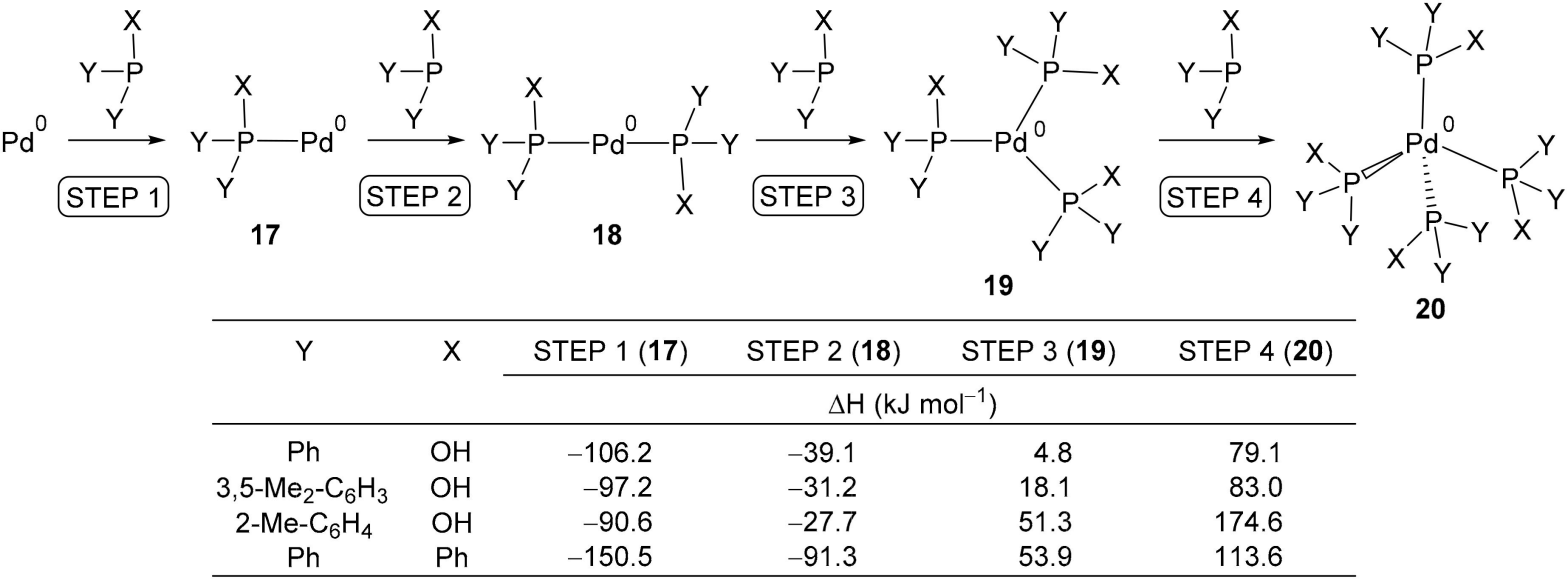
Consecutive ligation of “Pd(0)” by phosphines computed by the B3LYP/genecp//PCM(EtOH) method using the 6–31G(d,p) basis set for CHOP, and SDD(MWB28) for Pd atoms with the explicit-implicit solvent model.

In our calculations, the complexation of Pd(0) with diarylphosphine oxides with Ph-, 3,5-Me_2_-C_6_H_3_ and 2-Me-C_6_H_4_ substituents and triphenylphosphine was investigated (Scheme 9) [69]. It was found that the mono- (**17**) and bis-ligated Pd(0) complexes (**18**) may be formed in exothermic reactions (in STEP 1 and STEP 2, respectively) for all the cases investigated, as shown by delta enthalpy (Δ*H*) values of −150.5–90.6 kJ mol^−1^ and −91.3–27.7 kJ mol^−1^, respectively. The formation of tri-coordinated complex **19** from di-coordinated **18** is not as favorable with Ph_2_POH and (3,5-Me_2_-C_6_H_3_)_2_POH (Δ*H* = 4.8 and 18.1 kJ mol^−1^, respectively) as the bis-ligation. And what is more, with (2-Me-C_6_H_4_)_2_POH and PPh_3_, as a consequence of steric hindrance, the tris-ligation (STEP 3) becomes endothermic (Δ*H* = 51.3 and 53.9 kJ mol^−1^, respectively). In case of the (2-MePh)_2_POH and PPh_3_ ligands, the optimum is at bis-ligation, while with Ph_2_POH and 3,5-Me_2_-C_6_H_3_, the tris-ligation is optimal. The situation regarding enthalpies is rather similar for the tetra-ligation (**20**) [69]. The complex forming ability of the (2-Me-C_6_H_4_)_2_POH species is comparable with that of PPh_3_. It can be also said that while forms **18** and **19** are real complexes, **20** is dissociated variation. It is noted that the complexation status of a catalyst is a crucial in the reaction it is involved in. An “under-complexed” Pd is not active and not stabilized, hence, it may decompose and precipitate. At the same time, an overcrowded complex may not be active enough. From among the complexes discussed, **18** may be the best catalyst, **19** and **20** are hindered forms. It is noteworthy that Pd(PPh_3_)_4_ may also exist in the Pd(PPh_3_)_3_ + PPh_3_ form [70].

### 2.4. Experimental Results of the Effect of the Different Methyl Substituents in Diarylphosphine Oxides on the P–C Coupling Reaction with Bromobenzene

**Scheme 10:**
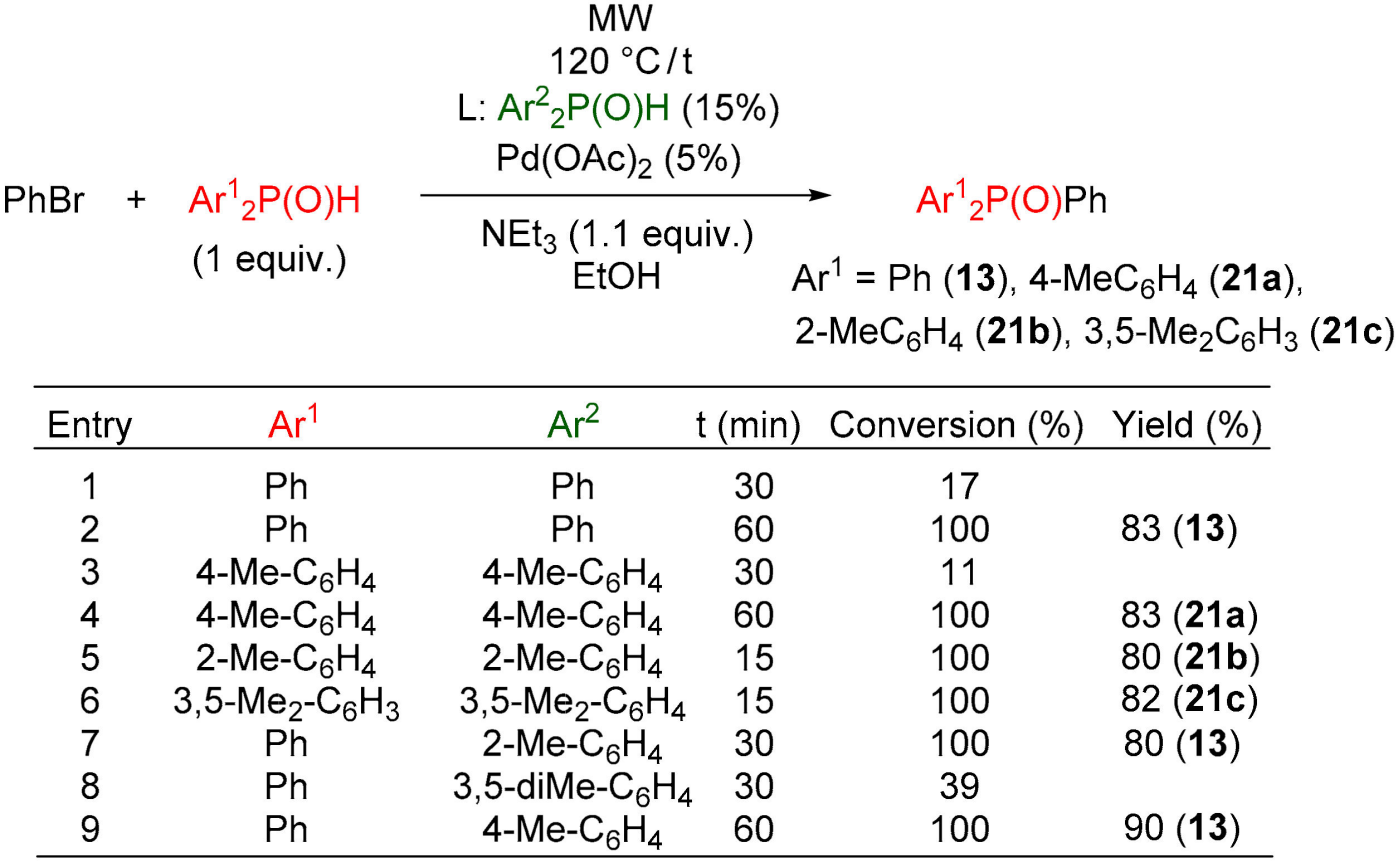
The Hirao reaction of PhBr with diarylphosphine oxides applying identical or different diarylphosphine oxide as the P-ligand.

The experimental data underlined the importance of the ligation [69]. In the preparative experiments, 1.15 equivalents of diarylphosphine oxides, such as Ph_2_P(O)H, (4-Me-C_6_H_4_)_2_P(O)H, (2-Me-C_6_H_4_)_2_P(O)H and (3,5-Me_2_-C_6_H_3_)_2_P(O)H were reacted with bromobenzene in the presence of 5% of Pd(OAc)_2_ and 1.1 equivalents of triethylamine in ethanol at 120 °C under MW irradiation. The results can be seen in Scheme 10. Under the conditions applied, completion of the Hirao reaction with Ph_2_P(O)H and (4-Me-C_6_H_4_)_2_P(O)H required 1 h (Scheme 10, entries 2 and 4). After 30 min, the conversions were only ≤17% (Scheme 10, entries 1 and 3). For the first sight, it was surprising that the similar reaction of (2-Me-C_6_H_4_)_2_P(O)H and (3,5-Me_2_-C_6_H_3_)_2_P(O)H took place much faster, both P–C couplings were complete after 15 min (Scheme 10, entries 5 and 6, respectively). From the efficient experiments, the corresponding diaryl-phenylphosphine oxides (**19a**–**d**) were obtained in yields of 80–83%.

The beneficial effect of the 2-Me or 3-Me group in the phenyl ring was concluded. To get further information, 0.15 equivalents of (2-Me-C_6_H_4_)_2_P(O)H was applied as the P-ligand in the Hirao reaction of Ph_2_P(O)H with bromobenzene that was carried out otherwise as above. As compared to the case, where Ph_2_P(O)H held the role of both the reagent and the preligand (Scheme 10, entries 1 and 2), the P–C coupling using (2-Me-C_6_H_4_)_2_P(O)H as the catalyst ligand was significantly faster, as a complete conversion required 30 min (Scheme 10, entry 7). The selectivity of the “mixed” Hirao reaction was surprisingly good, only 5% by-product (2-Me-C_6_H_4_)_2_P(O)Ph contaminated triphenylphosphine oxide. The catalyst formed from Pd(OAc)_2_ and (3,5-Me_2_-C_6_H_3_)_2_P(O)H as the P-ligand revealed an intermediate activity as compared to the complexes based on Ph_2_P(O)H or (2-Me-C_6_H_4_)_2_P(O)H as the preligands (Scheme 10, entry 8). The experiment applying (4-Me-C_6_H_4_)_2_P(O)H as the preligand gave similar result as the reaction using only Ph_2_P(O)H (Scheme 10, entry 9) suggesting that the 4-Me substituent has no effect on the course of the reaction. The results can be interpreted by assuming that the beneficial effect of (2-Me-C_6_H_4_)_2_P(O)H and (3,5-Me_2_-C_6_H_3_)_2_P(O)H is to promote the formation of a more active catalyst incorporating only two Ar_2_POH ligands. This is the consequence of steric hindrance due to the 2-Me group and 3-Me substituent, respectively. The activity of the Pd complexes with the P-ligands under discussion shows the following trend in respect of the P-species:

Ph_2_P(O)H ~ (4-Me-C_6_H_4_)_2_P(O)H < (3,5-Me_2_-C_6_H_3_)_2_P(O)H < (2-Me-C_6_H_4_)_2_P(O)H.

## 3. Catalysts for the Ni-Assisted Hirao Reactions

### 3.1. Theoretical and Experimental Studies on the Ni Catalyst

**Scheme 11:**
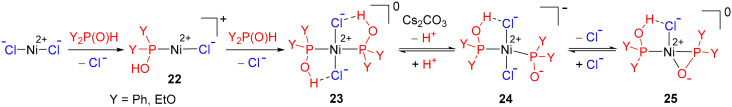
Mechanism for the formation of the active catalyst from NiCl_2_ and diphenylphosphine oxide or diethyl phosphite calculated by the B3LYP/6-31G(d,p)//PCM(MeCN) method.

As it was mentioned in the Introduction ( Section 1.2) not much is known about the exact nature of the Ni catalyst involved in the Hirao reaction. We investigated the route of the MW-assisted P–C coupling reaction of PhBr with diphenylphosphine oxide or diethyl phosphite applying NiCl_2_ as the catalyst precursor. In our case, the excess of the >P(O)H reagent served as the P-ligand. Preparative experiments suggested that two equivalents of the P-reagent to the NiCl_2_ was the optimum quantity [47,71]. Then, theoretical calculations suggested the following mechanism for the formation of the active catalyst entering in the Hirao reaction (Scheme 11) [71]. Two units of the >P(O)H reagent are attached to the central Ni atom of NiCl_2_ in the trivalent tautomeric form in a stepwise manner via intermediate **22**. The active catalyst (**25**) is developed after a consecutive deprotonation and the loss of a chloride anion, involving species **23** and **24**, respectively. This complex (**25**) will then participate in the oxidative addition with the aryl halide. It follows that, as a result, Ni(II) gets in the Ni(IV) state. This was indeed a fully surprising experience, but quantum chemical calculations confirmed this [71]. It was another surprise that in the “reductive” variation, again a Ni(II) → Ni(IV) oxidation was substantiated by the preparative experiments and calculations [72]. However, surveying the literature, the Ni(II) → Ni(IV) transition is well-known during the interconversion of different Ni complexes [73,74,75]. Moreover, a Ni(II) → Ni(IV) conversion was observed in the Ni-catalyzed alkylation of benzamides with alkyl halides [76].

### 3.2. A Theoretical Study on the Ligation of Ni(II)

**Scheme 12:**
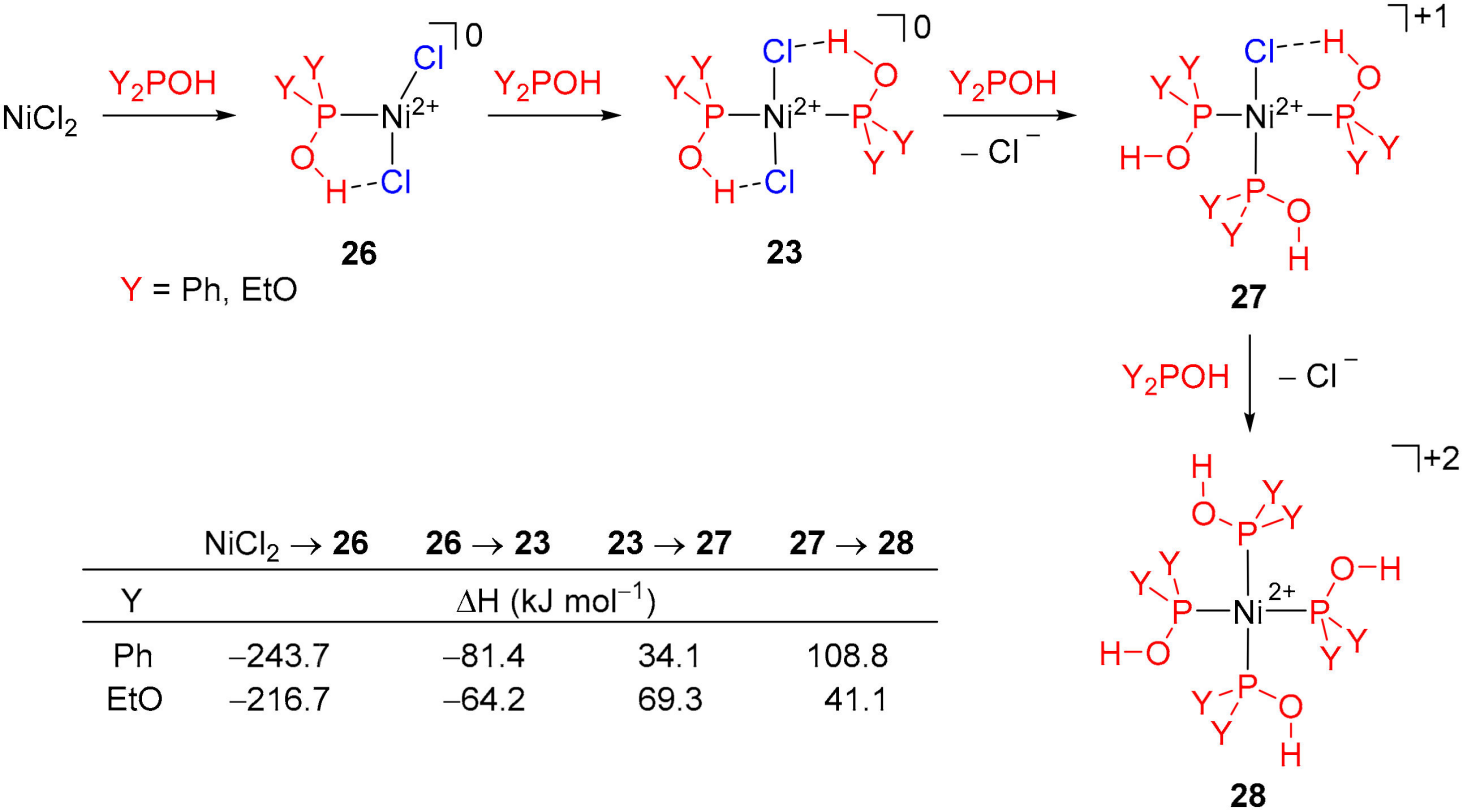
Ligation of NiCl_2_ with >P(O)H species together with complexation energies computed at B3LYP/6-31G(d,p)//PCM(MeCN) level of theory.

The consecutive complexation of NiCl_2_ with the trivalent tautomeric form (Y_2_POH) of diphenylphosphine oxide (Y = Ph) and diethyl phosphite (Y = EtO) was evaluated by quantum chemical calculations (Scheme 12). The endothermic tautomerization of Y_2_P(O)H to Y_2_POH (if Y = Ph, Δ*H* = 22.9 kJ mol^−1^, while if Y = EtO, Δ*H* = 25.1 kJ mol^−1^) [77] was also taken into consideration.

It was found that both the mono- and the bisligation was exothermic. Formation of complexes **26** and **23** can be characterized by enthalpy values of −243.7/−216.7 kJ mol^−1^, and −81.4/−64.2 kJ mol^−1^, respectively. At the same time, the tris-complexation leading to species **27** turned to endothermic (34.1/69.3 kJ mol^−1^). No wonder that the tetra-ligation leading to **28** is also unfavorable (108.8/41.1 kJ mol^−1^). It is noted that deprotonation of complexes **23**, **27**, and **28** is favorable characterized by enthalpy gains of −133.2/−171.4 kJ mol^−1^, −103.1/−115.1 kJ mol^−1^, and −476.7/−421.3 kJ mol^−1^, respectively. This ligation study confirmed that bis-complexation is the optimum in the cases under discussion. By the way, from the point of view of catalytic reactions, both an under-complexed metal (like in **26**) and overcrowded centrum (such as in **28**) are unfavorable.

## 4. Conclusions

Considering different variations, our experimental data and the theoretical calculations suggested that in the cases when Pd(OAc)_2_ was used as the catalyst precursor, “P_2_Pd(0)” was the initial species entering the catalytic cycle. Applying the excess of the Y_2_P(O)H reagent as the ligand, the catalyst is HOY_2_P–Pd(0)–PY_2_OH. Hence, considering that Y_2_P(O)H also serves as the reducing agent, three equivalents of the P-reagent to the Pd(OAc)_2_ precursor is needed. At the same time, when NiX_2_ is the catalyst precursor, no matter if there is a reducing agent or not, HOY_2_P–Ni(II)Cl–PY_2_O^–^ is the active catalyst. The involvement of Ni(II) instead of Ni(0) in the catalytic cycle is a brand new and surprising observation. Utilizing quantum chemical calculations mechanistic protocols were proposed for the formation of the “P_2_Pd(0)” and the “P_2_Ni(II)XccH” catalysts. Ligation studies also supported our findings.
